# Neurorestorative interventions involving bioelectronic implants after spinal cord injury

**DOI:** 10.1186/s42234-019-0027-x

**Published:** 2019-07-11

**Authors:** Newton Cho, Jordan W. Squair, Jocelyne Bloch, Grégoire Courtine

**Affiliations:** 1 École polytechnique fédérale de Lausanne (EPFL), Campus Biotech, Center for Neuroprosthetics and Brain Mind Institute, 1202 Genève, Switzerland; 2 Department of Neurosurgery, University of Toronto, Toronto, Ontario Canada; 3 Cumming School of Medicine, University of Calgary, Calgary, Canada; 4 MD/PhD Training Program, University of British Columbia, Vancouver, Canada; 5 Department of Neurosurgery, University Hospital of Lausanne (CHUV), Lausanne, Switzerland; 6 Defitech Center for Interventional Neurotherapies, EPFL / CHUV, Lausanne, Switzerland

**Keywords:** Spinal cord injury, Neuromodulation, Brain-computer interface, Electrical stimulation, Neurosurgery

## Abstract

In the absence of approved treatments to repair damage to the central nervous system, the role of neurosurgeons after spinal cord injury (SCI) often remains confined to spinal cord decompression and vertebral fracture stabilization. However, recent advances in bioelectronic medicine are changing this landscape. Multiple neuromodulation therapies that target circuits located in the brain, midbrain, or spinal cord have been able to improve motor and autonomic functions. The spectrum of implantable brain-computer interface technologies is also expanding at a fast pace, and all these neurotechnologies are being progressively embedded within rehabilitation programs in order to augment plasticity of spared circuits and residual projections with training. Here, we summarize the impending arrival of bioelectronic medicine in the field of SCI. We also discuss the new role of functional neurosurgeons in neurorestorative interventional medicine, a new discipline at the intersection of neurosurgery, neuro-engineering, and neurorehabilitation.

## Background

A century of medical research and clinical practice has transformed the management of patients with spinal cord injury (SCI). The standards of good clinical practice for a traumatic SCI consist of stabilizing spine fractures, decompressing the spinal cord, and maintaining optimal hemodynamics to avoid hypotension and secondary spinal cord damage. As soon as possible, the patient is transferred to a specialized SCI center where expert clinical teams deploy intensive rehabilitation programs and educate patients in the management of their bladder, bowel, and general body condition.

These surgical procedures, supportive measures, and rehabilitation programs have ameliorated neurological outcomes and decreased morbidity in patients with SCI (Fehlings et al. 2017). However, there is currently still no clinical trial that has reported robust efficacy of a spinal cord repair strategy for improving functional recovery after SCI. Due to the limited ability of the spinal cord for repair, many neurological deficits remain permanent, with devastating health consequences and substantial financial and social burdens for society. Until now, functional neurosurgeons are remotely involved in SCI medicine and their role remains confined to the management of spasticity or neuropathic pain with spinal cord stimulation.

Here, we summarize a series of preclinical and clinical advances in the development of neuromodulation therapies, brain-computer interfaces, and neurotechnology-supported neurorehabilitation programs that herald a new role of functional neurosurgeons in the restoration of neurological functions after SCI (Table 1).

**Table 1:** Summary of various bioelectronic interventions to improve neurologic function after spinal cord injury

Intervention	Type of data	Target/goal of intervention	Specific target(s) of action	Details of implant	Animal model/clinical trial	Results of studies	References
*Tonic electrochemical neuromodulation for hindlimb function*	Preclinical	*Infralesional* Improve hindlimb function	Lumbosacral cord	Stainless steel wires secured at midline over L2 and S1 to provide tonic epidural electrical stimulation (EES); 40 Hz s.c. or i.p. administration of pharmacologic agents	Rat: complete transection at T7 Rat: left lateral over-hemisection at T7 and right lateral hemisection at T10 Rat: severe contusion at T9 (250 kDyn) sparing < 10% tissue at lesion epicenter	EES + serotonergic agonists could generate weight-bearing leg movements as soon as 1 week after SCI Tonic electrochemical neuromodulation + daily training resulted in ability of rats to initiate and sustain full weight-bearing bipedal locomotion during electrochemical neuromodulation; recovery translated to other unpracticed tasks (i.e., swimming)	Courtine et al. 2009 Musienko et al. 2011 van den Brand et al. 2012 Asboth et al. 2018
	Clinical	*Infralesional* Improve leg function	Lumbosacral cord	16-electrode array implanted over midline of spinal cord segments L1-S1/2; pulse generator in abdominal pouch Stimulation parameters (frequency, amplitude) empirically driven via ad hoc observation	Chronic SCI patients (AIS A/B)	Intense locomotor training combined with epidural stimulation AIS B patients able to walk over ground with assistive devices and electrical stimulation; AIS A patients demonstrated some independent stepping on treadmill with body-weight support except one patient able to walk over ground and independently stand during stimulation	Angeli et al. 2018 Gill et al. 2018
*Tonic electrical neuromodulation for autonomic function*	Clinical	*Infralesional* Improve autonomic function	Lumbosacral cord	16-electrode array implanted at T11-L1 vertebral levels over spinal cord segments L1-S1 Parameters of stimulation optimized empirically	Chronic SCI patients (AIS A/B)	Reduced blood pressure drop with orthostatic stress test (transitioning from supine to sitting) with EES Resolution of orthostatic-induced symptoms (i.e., dizziness, poor concentration) and prevention of decrease in MCA blood flow Persistent hypotension evident in some patients resolved with EES	West et al. 2018 Aslan et al. 2018 Harkema et al. 2018a Harkema et al. 2018b Darrow et al. 2019
*Multi-directional robotic gravity assist*	Preclinical/Clinical	Improve locomotor ability by facilitating training	N/A	Multidirectional robotic support system; three translational axes in Cartesian frame and one rotational axis; suspension system fabricated with spring assembly to decouple inertia of robotic structure from subject Real-time control of propulsion, lateral balance, and body-weight support along four degrees of freedom	Rat: cortical stroke, moderate, and severe SCI Human: stroke, SCI, normal subjects	Enabled skilled motor control after stroke and coordinated locomotion on staircase after moderate (lateral hemisection) and severe SCI (staggered lateral hemisection) in rats Human gravity-assist algorithm: supervised machine learning approach that predicted optimal upward support forces for each patient based on collected kinematic variables; simulations guided personalization of forward force for patient-specific needs Algorithm optimized upward and forward forces to facilitate locomotion depending on patient needs	Dominici et al. 2012 Mignardot et al. 2017
*Spatiotemporal electrical stimulation paradigms*	Preclinical	*Infralesional* Improve hindlimb function	Lumbosacral cord	Epidural implant fabricated with UV photolithographic patterning of photosensitive polyimide; microelectroforming to create gold electrodes and embedded gold interconnects; contact interface over-molded with thin layer of medical grade silicone to improve biointegration	Rat: complete transection T8 Rat: dorsal contusion T9	Delivery of stimulation at spatial “hot spots” (motor pools innervating different hindlimb muscles) for flexion and extension in the cord Closed-loop stimulation delivered based on angular displacement of hindlimb endpoint around its center of rotation Spatiotemporal neuromodulation gait patterns closer to intact rats than with continuous stimulation after SCI	Wenger et al. 2016
	Clinical	*Infralesional* Improve leg function	Lumbosacral cord	16-electrode paddle array implanted over lumbosacral cord segments; connected to pulse generator in abdomen Rostro-caudal positioning of electrode array optimized based on EMG responses to single-pulse EES intra-operatively	Chronic SCI patients (AIS C/D)	Simulations based on patient MRI and CT scans of the spine guided identification of optimal electrode configurations leg muscle recruitment Closed-loop triggering of EES based on foot trajectory Spatiotemporal EES enabled overground locomotion within one week; patients able to increase step elevation 3- to 5-fold when asked, during EES delivery Continuous EES enhanced muscle activity but poorly facilitated overground locomotion	Wagner et al. 2018
*Brain-computer interface*	Preclinical	Improve hindlimb function	M1; lumbosacral cord	Rat: 32-channel microelectrode array in layer V of leg region of right motor cortex Wire electrodes sutured to dura over dorsal aspect of L2 and S1 to deliver EES (tonic, 40 Hz) Rhesus monkey: 96-channel microelectrode array implanted into M1; custom-made spinal implant (see “Spatiotemporal electrical stimulation paradigms”) inserted into T13-L1 vertebral level; decoded swing and stance from neural activity and triggered stimulation protocols wirelessly	Rat: dorsal contusion at T9-T10 (250 kDyn) Rhesus monkey: lateral CST lesion T7/8	Rat proportional BSI: Normalized cumulative firing in motor cortex resulted in delivery of stimulation burst over electrode at L2 (amplitude based on linear relationship) Compared to continuous stimulation, proportional BSI enabled rats to produce gait patterns resembling intact rats and also resulted in better locomotor performance with rehabilitation Within 1 week post-SCI and without training, BSI in monkey restored weight-bearing locomotion on treadmill and overground	Bonizzato et al. 2018 Capogrosso et al. 2016
*Brain-computer interface*	Clinical	Improve upper limb movement	M1 with prosthetic limb	Microelectrode array implanted in M1 to decode motor intention based on neural spiking activity Movement of prosthetic limb (i.e., DLR Light-Weight Robot III) based on decoded motor intention	Chronic tetraplegia secondary to brainstem stroke, spino-cerebellar degeneration	Subjects able to use robotic arm to reach and grasp foam ball targets; able to grasp bottle and drink coffee through a straw Able to control prosthetic limb freely in 3D space and after training, perform coordinate reach and grasp movements	Hochberg et al. 2012 Collinger et al. 2013
	Clinical	Improve upper limb movement	M1 with neuro-muscular electrical stimulator (NMES)	Microelectrode array implanted in M1; subject trained to use motor cortical neuronal activity to control NMES, which delivers electrical stimulation to arm muscles via percutaneous electrodes	Chronic tetraplegia secondary to SCI	Regained volitional movement via intracortical signals linked to neuromuscular stimulation in real time Able to perform grasping of bottle, pouring into a jar, and stirring with a stick; drinking mug of coffee and feeding self with paralyzed arm	Bouton et al. 2016 Ajiboye et al. 2017
	Clinical	Improve upper limb sensation	S1	Microelectrode array implanted in S1, wired to external connector attached to skull	Chronic tetraplegia secondary to SCI	Intracortical microstimulation evoked sensations with projected fields in the fingers Using Modular Prosthetic Limb, increase in motor torque when limb touched linearly converted to stimulation amplitude; subject able to identify the finger touched	Flesher et al. 2016
*Deep brain stimulation*	Preclinical	*Supra-lesional* Improve hindlimb function	MLR	000-gauge stainless steel needle soldered to screw connector implanted stereotactically in MLR and secured with dental cement to the skull	Rat: incomplete SCI with scalpel blade and iridectomy scissors	Increasing stimulation intensity resulted in rat walking to galloping and increase in swimming speed in intact animals 4 weeks after SCI, increase in walking speed with increase in MLR stimulation intensity; reduction in paw drag	Bachmann et al. 2013
	Preclinical	*Supra-lesional* Improve hindlimb function	NRM	Microelectrode implanted stereotactically in the NRM; programmed to give 5 min of 8 Hz stimulation alternated with 5 min of rest for 12 daytime hours followed by 12 h of rest	Rat: contusion T8	Reduction of mechanical allodynia in forepaws 6 weeks after injury; reduction in astrogliosis at 15 weeks in the spinal cord	Hentall and Burns 2009
*Vagal nerve stimulation*	Preclinical	*Supra-lesional* Improve forelimb function	Vagus nerve	Vagus nerve cuff electrode placed around left cervical branch of vagus nerve; closed-loop delivery of stimulation on trials in which pull forces of rat forelimb fall within the top quintile of previous trials	Rat: right (200 kDyn) or midline (225 kDyn) C6 dorsal contusion	Compared to rehabilitation alone, closed-loop VNS stimulation significantly improved recovery of forelimb strength	Ganzer et al. 2018
*Motor cortex stimulation*	Preclinical	*Supra-lesional* Improvement of limb function	Corticospinal tract	Electrode insertion for stimulation of pyramidal tract or motor cortex	Rat: unilateral pyramidal tract lesion	Continuous stimulation for 10 days significantly augmented strength of ipsilateral motor responses (recorded in the deep radial nerve); increase in density of corticospinal tract projections	Carmel and Martin 2014
	Clinical	*Supra-lesional* Improvement of limb function	M1	Repetitive transcranial magnetic stimulation of M1; frequency ranging between 5 and 20 Hz for between 5 and 15 sessions	Subacute and chronic SCI patients (AIS A-D)	Limited and variable improvements in sensory and motor function	Tazoe and Perez 2015

*AIS* American Spinal Injury Association Impairment Scale, *i.p.* intraperitoneal; *BSI* Brain-spine interface, *EES* Epidural electrical stimulation, *EMG* Electromyogram, *M1* Primary motor cortex, *MCA* Middle cerebral artery, *MLR* Mesencephalic locomotor region, *NMES* Neuromuscular electrical stimulator, *NRM* Nucleus raphe magnus, *S1* primary sensory cortex, *s.c.* subcutaneous

### The era of restorative neurosurgery

The brain broadcasts movement-related commands through parallel neuronal pathways that cascade from the cortex and brainstem to executive centers residing in the spinal cord (Arber and Costa 2018). An SCI scatters this exquisitely-organized communication system, which results in severe motor deficits and alters critical physiological functions. However, most SCIs spare bridges of intact neural tissue that contain fibers still connected to executive centers located below the injury. For unclear reasons, these anatomically intact neural projections remain functionally silent. Moreover, the vast majority of circuits involved in producing movements and regulating physiological functions are distant from the spinal cord damage. Consequently, the anatomical integrity of these circuits is not compromised. This understanding has triggered the development of engineering interventions that tap into residual projections and spared circuits to enable the control of movements, regulate physiological functions, and improve neurological recovery.

**Fig. 1:**
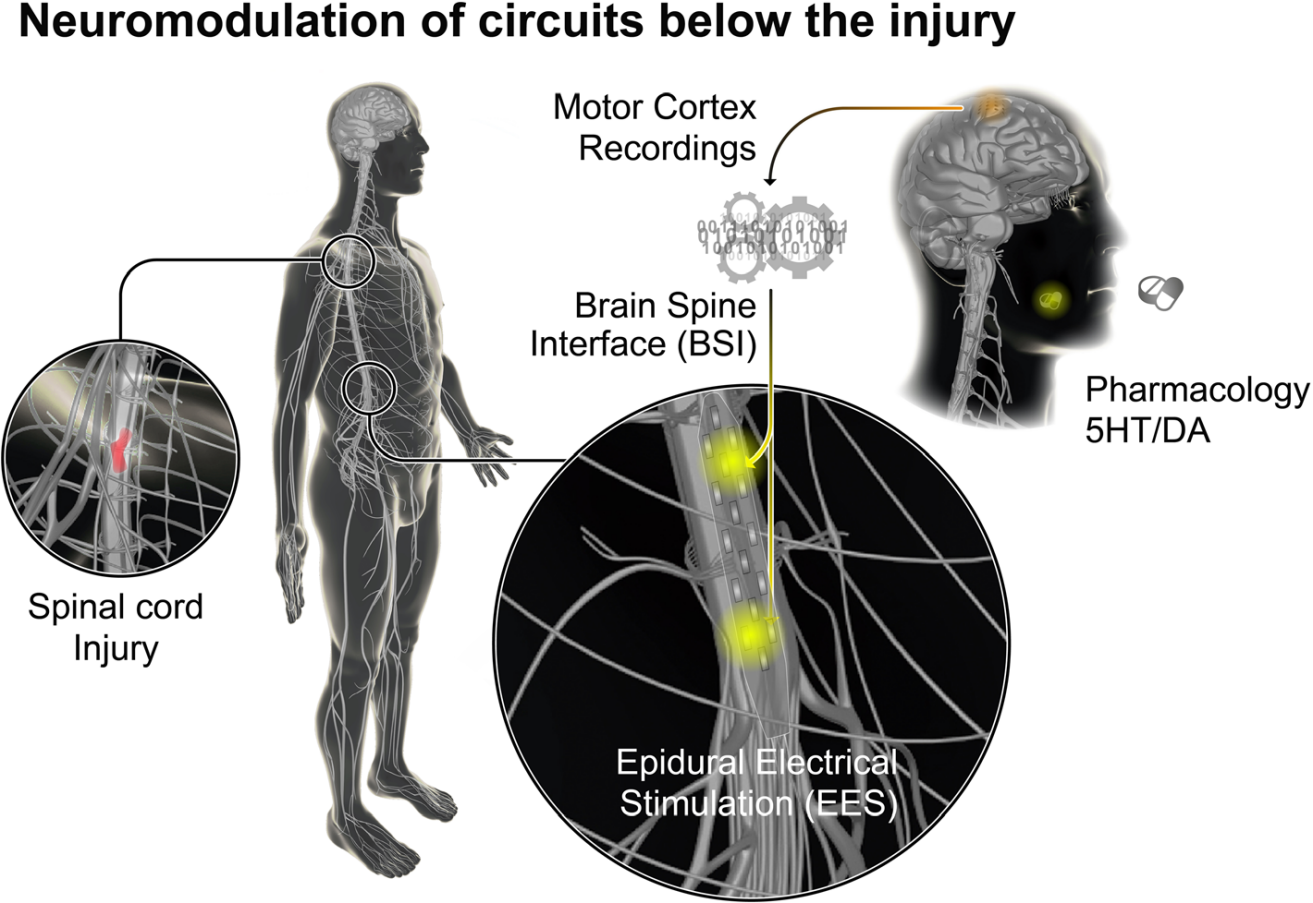
Neuromodulation strategies to engage circuits below the lesion after SCI. The reactivation or modulation of spinal circuits for locomotion has been demonstrated with the use of epidural electrical stimulation (EES) combined with the oral or intrathecal administration of serotonergic and dopaminergic agonists. EES can also be used to optimize autonomic function post-SCI (i.e., blood pressure management). Brain-spine interfaces (BSIs) also provide an alternative strategy for locomotion through bypassing the injury

**Fig. 2:**
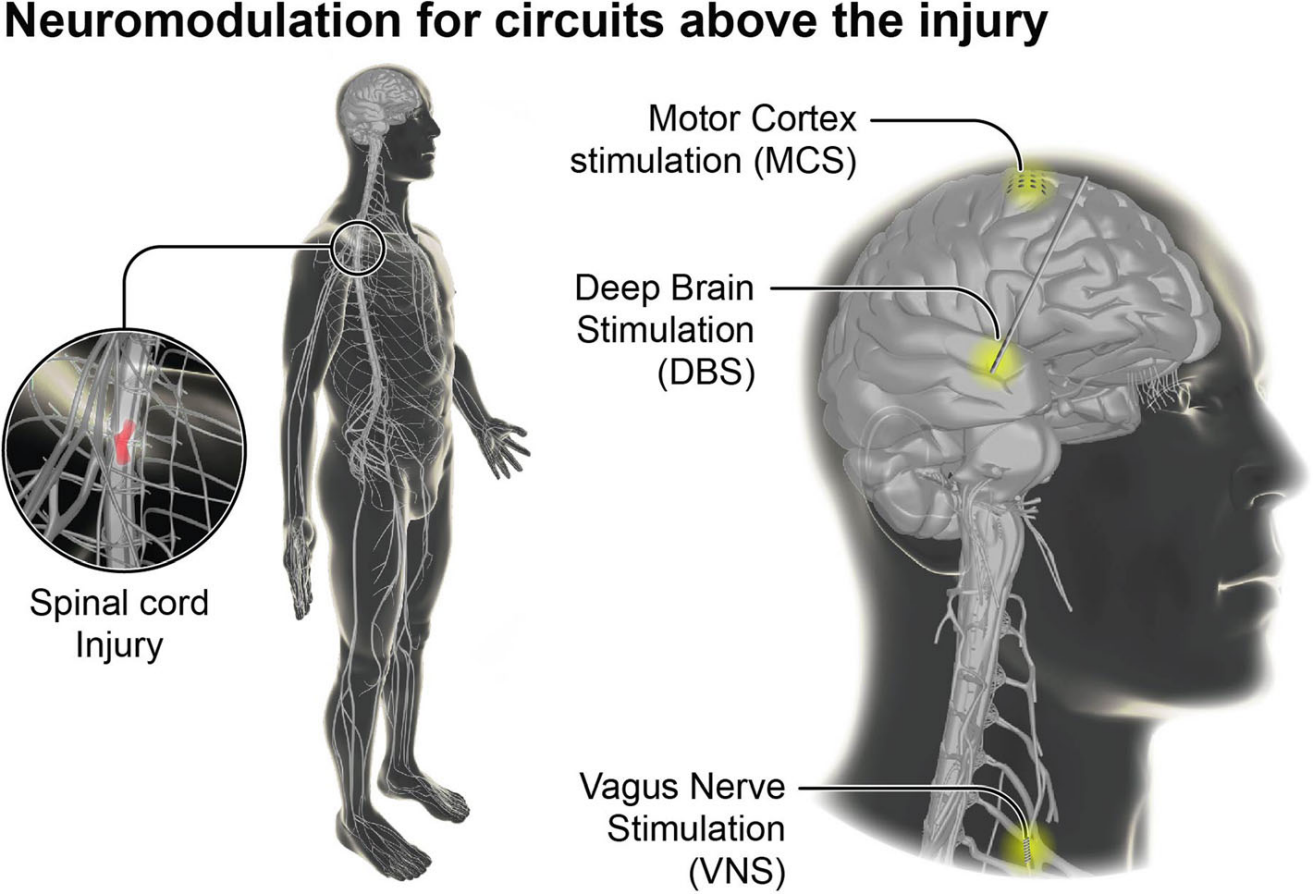
Neuromodulation strategies to engage circuits above the lesion after SCI. Neuromodulation therapies have been delivered to the mesencephalic locomotor region (MLR) using deep brain stimulation (DBS) in order to facilitate locomotion. Motor cortex stimulation (MCS) has been applied for extensive periods of time daily to promote the growth and sprouting of corticospinal tract fibers. Finally, vagus nerve stimulation (VNS) has been applied to augment motor learning and plasticity during motor rehabilitation

These interventions all have in common the surgical implantation of bioelectronic devices connected to electrode arrays in order to record from neural ensembles or deliver electrical stimulation. Bioelectronic treatments focusing on the delivery of electrical stimulation are a type of neuromodulatory therapy. These stimulation-based neuromodulation therapies target circuits that can be located below the injury (infralesional, Fig. 1) or at different levels above the injury (supralesional, Fig. 2). The simplest approach involves the delivery of continuous stimulation over broad regions of the brain, midbrain and spinal cord, or even to peripheral nerves. However, the identification of the mechanisms through which electrical stimulation paradigms modulate circuits have led to more effective stimulation protocols that are modulated in the temporal and/or spatial domains. The conception of neuromodulation therapies that are directly controlled via brain signals is also emerging quickly.

All these treatments involve the contribution of a functional neurosurgeon who not only needs to implant one or several bioelectronic devices, but also must interact effectively with multidisciplinary teams of engineers, neurologists, and physical therapists in order to deploy these treatments. Below, we summarize the scientific basis and technological framework of each of these bioelectronic treatments, and envision the steps forward to turn current proofs of concepts into widely available medical treatments for SCI.

## Targeting circuits below the SCI: infralesional neuromodulation therapies

### Reactivating spinal circuits involved in producing movement

The specialized features of locomotor-related descending commands originating from the brainstem remain vividly debated and studied. However, their functional contribution can be (over) simplified into two main functions: provide monoaminergic modulation and glutamatergic excitation. The interruption of descending pathways from the brainstem thus deprive spinal circuits from these essential sources of modulation and excitation. While executive centers residing in the spinal cord are intact, they fail to produce leg movements. This understanding triggered the development of neuromodulation therapies that seek to replace these missing sources of modulation and excitation to reactivate spinal circuits, and thus enable motor control.

Preclinical research in mammal models showed that pharmacological agents and electrical spinal cord stimulation were highly effective to reactivate executive spinal circuits involved in leg motor control. The pharmacological agents can target a broad range of serotonin, dopamine and noradrenaline receptor subtypes that each modulate specific features of movement such as weight bearing capacities or inter-limb coordination (Musienko et al. 2011; Rossignol et al. 2001). The most effective pharmacological interventions targeted 5HT_1A_, 5HT_2A/C_ and 5HT_7_ receptors subtypes—for example with Quipazine and 8-OHDPAT (Courtine et al. 2009). Direct spinal cord stimulation has been achieved with invasive and noninvasive neurotechnologies that include intraspinal stimulation/epidural electrical stimulation and transcutaneous electrical stimulation/magnetic stimulation, respectively (Gerasimenko et al. 2015; Wenger et al. 2016; Grahn et al. 2017; Angeli et al. 2015; Herman et al. 2002; van den Brand et al. 2012; Danner et al. 2015; Minev et al. 2015; Holinski et al. 2016; Zimmermann et al. 2011; Kasten et al. 2013; Angeli et al. 2014; Lu et al. 2016). Thus far, epidural electrical stimulation (EES) applied over the dorsal aspect of the spinal cord has been the most promising paradigm to engage lumbosacral circuits. Even in the complete absence of supraspinal input, the administration of serotonergic agonists and continuous EES enabled the immediate production of complex motor behaviors. Mice, rats, and cats with complete mid-thoracic transection were thus able to stand and walk over a broad range of speeds and directions while supporting their body weight (Courtine et al. 2009; Dominici et al. 2012). Under these conditions, task-specific sensory information arising from the legs becomes the source of modulation that governs the production of movement (Fong et al. 2009).

When the interruption of descending pathways is complete, these movements remain involuntary. However, studies in incomplete rodent models of SCI showed that a small percentage of spared fibers is sufficient to reestablish voluntary control of executive centers in the lumbosacral spinal cord. For example, after a severe contusion SCI that spares less than 10% of white matter tracts, the delivery of pharmacological and electrical neuromodulation therapies instantly enabled graded cortical control over the degree of leg extension during locomotion (Asboth et al. 2018). Since these contusions abolish all corticospinal tract synaptic projections below the injury, the cortical command cannot be conveyed directly to the lumbosacral spinal cord. Indeed, optogenetic and chemogenetic manipulations demonstrated that glutamatergic projection neurons located in the ventral gigantocellular nucleus (reticular formation) relay the cortical command to the spinal cord (Asboth et al. 2018). The ubiquitous location of reticulospinal fibers in the white matter ensures that a subset of these projections are spared, regardless of the inherently variable location of spinal cord damage. It is important to understand that in the absence of spinal cord neuromodulation therapies, these spared descending fibers are functionally silent. They fail to elicit any detectable muscle contraction. Neuromodulation therapies thus amplify the residual commands from the brain. In these conditions, executive centers in the spinal cord process supraspinal and sensory information in order to integrate volition into the execution of movements that are continuously adapted to the requirements of the performed tasks.

Studies in preclinical models of SCI evolved in parallel to multiple case studies conducted in humans with incomplete or complete SCI. To modulate the spinal cord electrically, scientists used single leads or paddle electrode arrays implanted over the lumbar spinal cord that they interfaced with implantable pulse generators commonly used in pain treatments. Studies from multiple independent laboratories thus showed that the delivery of continuous electrical stimulation (tonic) over the lumbar spinal cord immediately reestablished intentional control over the activity of previously paralyzed leg muscles, even more than a decade after the occurrence of the SCI. Continuous EES also restored full weight-bearing standing and facilitated stepping (Angeli et al. 2018; Gill et al. 2018). It was also shown that monoaminergic agonists could amplify the facilitation of movement mediated by electrical spinal cord stimulation (Gerasimenko et al. 2015). These clinical studies confirmed the validity of the concepts established in preclinical models, which triggered a surge of interest for the development of neurotechnologies that are optimized for motor-related applications.

The combination of finite element modeling (FEM) of electrical spinal cord stimulation with anatomically realistic models of the main afferent and efferent circuits located in the spinal cord revealed that the electrical fields elicited by EES do not penetrate the spinal cord (Capogrosso et al. 2013; Rattay et al. 2000). Consequently, EES does not modulate motor neurons directly. The electrical current flows around the spinal cord within the cerebrospinal fluid (CSF) where it activates the neural structures with the lowest impedance. The large-diameter proprioceptive afferent fibers are the least resistive neural elements in this region. Therefore, EES depolarizes proprioceptive afferent fibers at their entrance in the spinal cord, where they exit the posterior roots. The extensive branches of proprioceptive fibers in the spinal segments rostral and caudal to their entrance lead to a broad increase in the excitability of spinal circuits (Edgerton et al. 2008; Gerasimenko et al. 2007; Ichiyama et al. 2008; Musienko et al. 2012). In addition, each afferent volley leads to the trans-synaptic activation of motor neurons through the recruitment of proprioceptive feedback circuits (Dy et al. 2005; Lavrov et al. 2008a; Lavrov et al. 2008b). Concretely, each pulse of EES gives rise to monosynaptic and polysynaptic motor responses, the succession of which contributes to elaborating the activity of leg muscles (Wenger et al. 2016; Capogrosso et al. 2013; Capogrosso et al. 2018; Moraud et al. 2016). EES frequency determines how frequently proprioceptive feedback circuits are recruited, and thus how much activity is elicited in leg muscles (Wenger et al. 2014).

**Fig. 3:**
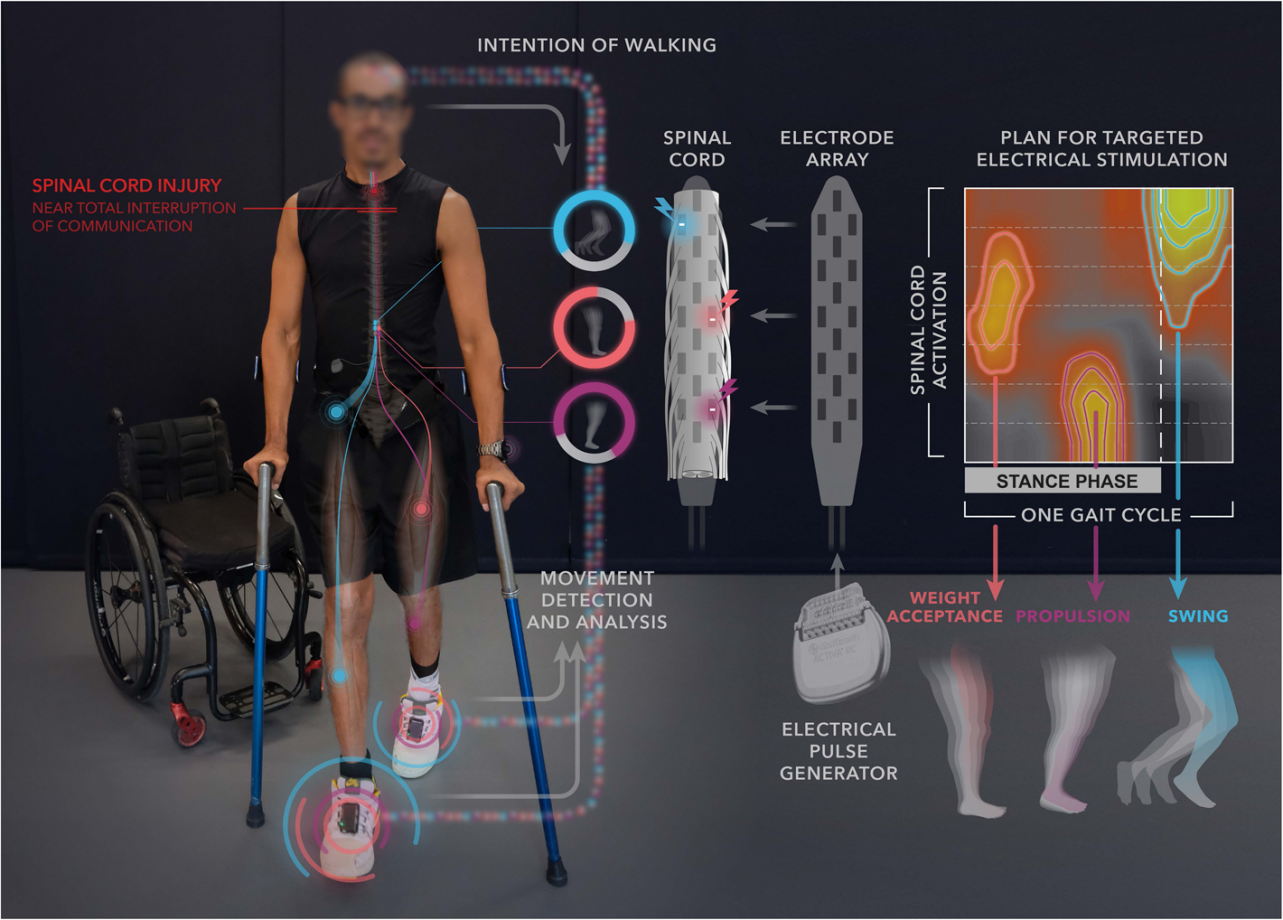
Spatiotemporal EES reproduces the natural activation of the spinal cord. Delivery of EES bursts matching the spatial and temporal dynamics of natural motor neuron activation immediately enables locomotion after SCI. Decoding algorithms detect foot movements in order to adjust the location and timing of the spatiotemporal stimulation sequences to the current needs of the patient. The spinal cord activation map is reconstructed based on the projection of electromyographic recordings onto the theoretical location of motor neurons in the spinal cord

This understanding led to a paradigm shift in the design of stimulation protocols (Capogrosso et al. 2018). The reasoning was the following: if motor neurons are engaged indirectly through the recruitment of proprioceptive afferents located in the posterior roots, then targeting individual posterior roots would provide access to the motor neuron pools located in the spinal segment innervated by each root. These predictions have been verified consistently in rodent (Wenger et al. 2016) and nonhuman primate models (Capogrosso et al. 2016), and more recently in humans (Wagner et al. 2018). This spatial selectivity suggested that the delivery of spatially-selective trains of EES with a timing reproducing task-dependent activation of motor neuron pools would result in a more robust and more physiological activation of the spinal cord during movement execution (Fig. 3). This spatiotemporal neuromodulation strategy restored full weight bearing locomotion in rats with complete SCI, which was not possible with continuous EES (Wenger et al. 2016). Since the recruitment of motor neuron pools with EES was restricted to the phase during which they were active, the amplitude and frequency of EES could be manipulated over a broad range of values. This large parameter space allowed the control of leg muscle activity with high precision. A simple tuning of EES amplitude or frequency enabled a precise adjustment of the extent of flexion and extension movements. For example, real-time control of EES parameters allowed rats with complete SCI to climb up staircases of various heights and lengths with fluidity (Wenger et al. 2016; Wenger et al. 2014).

Translation of this spatiotemporal stimulation strategy in humans required upgrading an implantable pulse generator commonly used for deep brain stimulation therapies with wireless modules that enabled real-time control over the location and timing of multiple concomitant EES bursts (Fig. 3). The pulse generator was connected to a paddle electrode array used for pain therapies. Since the configuration of the electrodes was not tailored for motor-related applications, the surgical positioning of the array was critical. Before surgery, a personalized computational model of the lumbosacral spinal cord was elaborated from a high-resolution MRI scan for each patient. Computer simulations guided the neurosurgeon in the positioning of the array, which was fine-tuned based on electrophysiological recordings (Wagner et al. 2018).

**Fig. 4:**
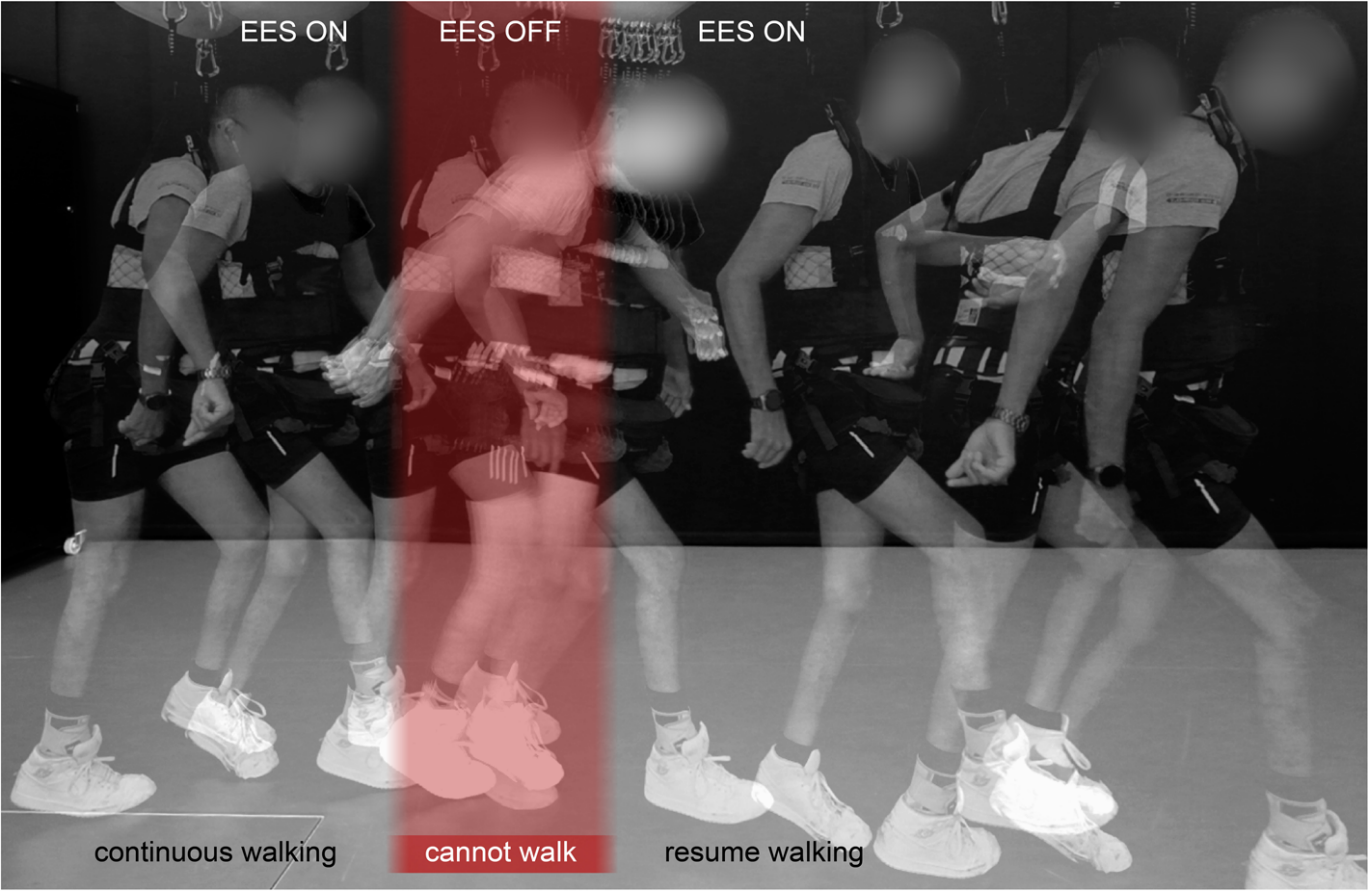
Chronophotography illustrating the recovery of locomotion during targeted EES. The patient is receiving targeted EES while suspended in a cutting-edge body-weight support system. EES is switched on and off, showing that the recovery of overground locomotion only occurs during EES

The delivery of EES bursts matching the spatial and temporal dynamics of natural motor neuron activation led to an immediate recovery of locomotion. Within 5 days, all tested individuals who had sustained a severe SCI several years prior to the surgical intervention were able to produce weight-bearing, independent stepping movements on a treadmill and overground (Fig. 4). Instead, continuous EES was poorly effective in these participants due to the cancellation of proprioceptive information that occurs during continuous EES in humans (Formento et al. 2018). Spatiotemporal stimulation paradigms mitigate the cancellation of proprioceptive information, since afferent populations are recruited transiently and in phase with the movement they encode.

After 1 month of tuning and habituation to the stimulation, individuals who were not able to take independent steps without stimulation exhibited coordinated locomotion for duration as long as 1 hour, covering up to 1 kilometer in 1 hour without external assistance. During stimulation, they were able to modulate the activity of previously paralyzed muscles voluntarily in order to produce three to five-fold increases in their step elevation or adjust their stride length to increasing treadmill belt speeds.

The development of next-generation spinal cord neuromodulation therapies required a series of technological advances, both in preclinical models and for clinical applications. These innovations included novel spinal implants, real-time control infrastructures, upgraded firmware for pulse generators, personalized computational models and spatiotemporal stimulation algorithms (Wenger et al. 2016; Minev et al. 2015; Capogrosso et al. 2013; Capogrosso et al. 2018; Moraud et al. 2016; Wenger et al. 2014; Capogrosso et al. 2016; Courtine and Bloch 2015). Functional neurosurgeons played a critical role in these early developments. They will continue contributing to designing and optimizing next-generation neurotechnologies that will be uniquely tailored to the requirements of motor-related applications.

### Intense training enabled by spinal cord neuromodulation therapies

Experiments conducted in the 1980s showed that cats with complete SCI could regain independent stepping when they were trained intensively on a treadmill with manual assistance (de Leon et al. 1998). They also regained the ability to stand for several minutes to hours when they were trained for this task (De Leon et al. 1998). However, they then lost the ability to step. These unexpected results showed that the spinal cord could learn a task that was performed regularly, and that task-specific training altered the anatomical and functional connectivity of the trained spinal circuitry (Tillakaratne et al. 2002; Ichiyama et al. 2011). These results compelled many specialized rehabilitation centers to develop procedures to train paralyzed patients to step on a treadmill with manual assistance—yet, with disappointing outcomes (Dietz et al. 1994). In humans, the excitability of the spinal cord appeared too depressed after SCI to enable the coordinated recruitment of motor neuron pools during passive leg movements (Harkema 2001). Consequently, activity-dependent plasticity was as limited as the amount of activity elicited within the sensorimotor circuitry (Field-Fote 2015).

This understanding suggested that it was critical to enable robust levels of activity during rehabilitative training to steer activity-dependent plasticity in the trained circuitry (Edgerton et al. 2008). During the same period, pharmacological and electrical neuromodulation of the spinal cord had shown the ability to enable stepping in rat models of SCI (Courtine et al. 2009; Ichiyama et al. 2008). The next logical step was to facilitate step training with these neuromodulation therapies. Intense rehabilitative training enabled by neuromodulation therapies induced dramatic improvements of motor capacities. Rats with severe SCI leading to permanent leg paralysis regained the ability to transform environmental cues into specialized motor commands that allowed them to walk overground, climb up a staircase and even swim (van den Brand et al. 2012; Asboth et al. 2018). The systematic dissection of the anatomical and functional mechanisms revealed that the motor cortex orchestrated the recovery, regardless of the specific descending tracts that were spared. In all the studied injury models, it was found that the motor cortex developed new routes involving neuronal relays in the brainstem and/or within bridges of intact tissues in the spinal cord (van den Brand et al. 2012; Asboth et al. 2018). These indirect neuronal pathways were sufficient to transfer task-specific motor cortex commands past the injury to the executive centers located in the spinal cord that produce leg movements. Importantly, this anatomical and functional reorganization did not take place when rats were trained to step automatically on a treadmill (van den Brand et al. 2012). Critical to trigger the plasticity of descending pathways was a cutting-edge multidirectional robotic body weight support system that positioned the rats bipedally (Dominici et al. 2012). This posture forced them to send motor commands to their leg muscles to propel their body forward toward a food reward. Under these training conditions, rats regained supraspinal control over previously paralyzed muscles even without the need of neuromodulation (Asboth et al. 2018). This neurological recovery highlighted the importance of goal-directed training to promote activity-dependent plasticity throughout the locomotor circuitry.

Clinical studies confirmed these results in humans with SCI. The first clinical studies were conducted using continuous (tonic) EES. Two patients with motor complete SCI but partially preserved sensory function followed intense locomotor training for more than 1 year. Both recovered the ability to walk overground with assistive devices during continuous EES (Angeli et al. 2018). However, they did not show improvement in neurological function. The two other patients in this trial exhibited a functionally complete SCI. Both patients achieved some independent stepping on the treadmill with bodyweight support and manual assistance (Angeli et al. 2018). In a second independent study, one patient with complete paraplegia could step overground with a front wheel walker and assistance from therapists (Gill et al. 2018).

**Fig. 5:**
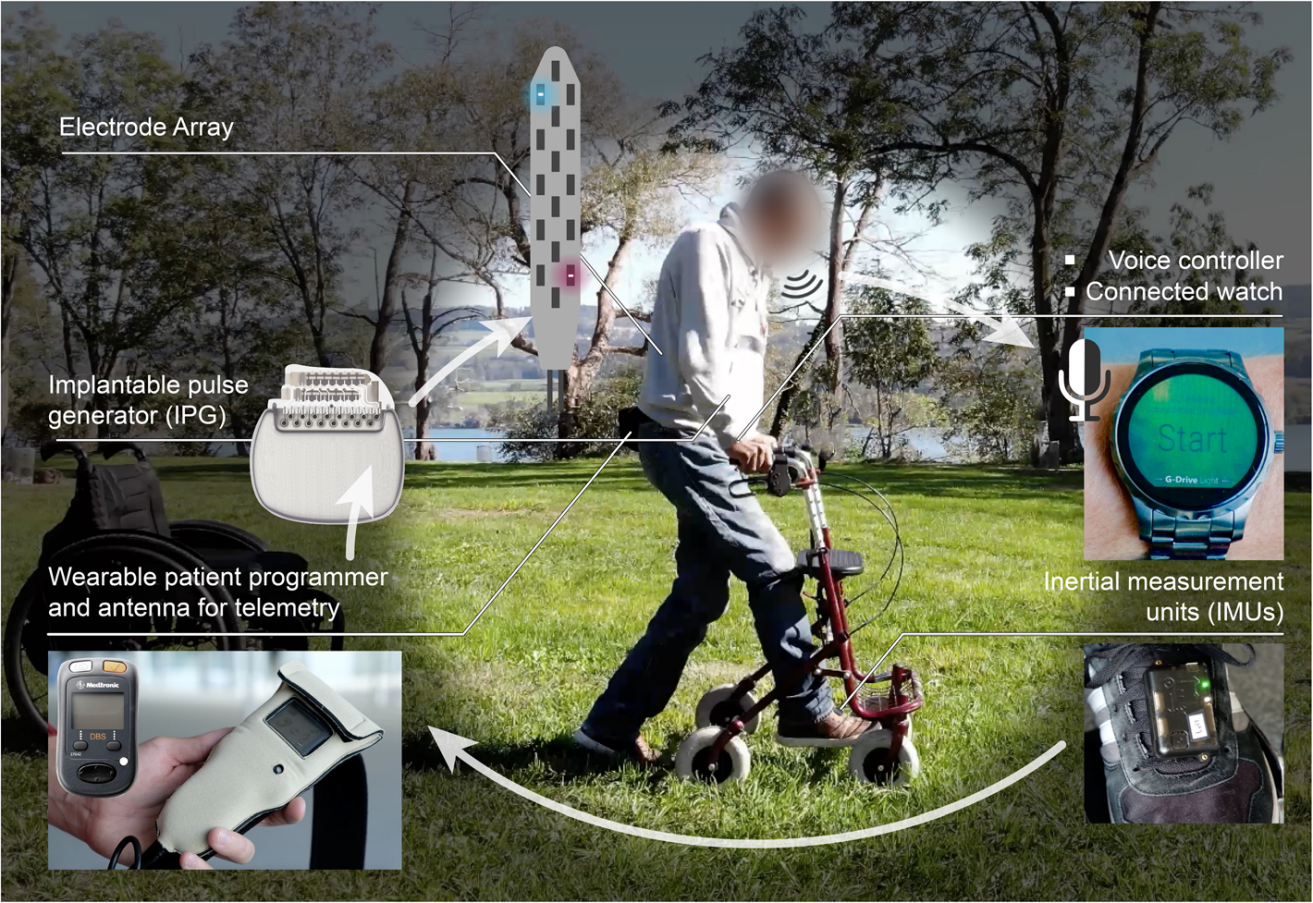
Ecological principles should guide the development of bioelectronic technology for SCI. Making the technology useable in the patient’s natural setting is paramount to its utility. This photograph illustrates the technological framework that enables real-time control of EES outside the laboratory environment. Developing technology based on these ecological principles will require the input and collaborative effort of multiple specialties including neurosurgeons, neurologists, rehabilitation specialists, physiotherapists, engineers, and scientists in order to make bioelectronic technology for patients with SCI safe and user-friendly

The most recent study sought to reproduce the therapeutic conditions that mediated the more pronounced functional recovery in preclinical models of SCI, as described above. This involved the conception of a multidirectional robotic body weight support system that allows patients to walk naturally in a large workspace. A gravity-assist algorithm personalized the amount of forces applied to the trunk in order to establish natural interactions between gravitational forces and gait dynamics while providing the optimal body weight support to the patient (Mignardot et al. 2017). Three patients followed an intensive gait training program enabled by this gravity-assist and spatiotemporal neuromodulation of the lumbosacral spinal cord (Wagner et al. 2018). All three patients could not ambulate or were completely paralyzed prior to their enrollment, despite their involvement in extensive rehabilitation programs. After less than a month of training, all participants were able to walk overground during stimulation. Locomotor performance improved dramatically over the course of the 5 months of training. During stimulation, they regained the ability to walk long distances in ecological settings using assistive devices (Fig. 5). For this purpose, they wore inertial measurement units (IMU) attached to their feet. Decoding algorithms processed these signals to detect foot movements and thus adjust spatiotemporal stimulation sequences to the current needs of the patients (Capogrosso et al. 2018). A watch responding uniquely to their own voice allowed them to switch the stimulation on and off. While this treatment paradigm remains at the stage of a proof of concept, it is worth noting that ecological principles guided its conceptual and technological design. Such ecoprosthetic designs should be encouraged more systematically for the development of neurotechnologies (Courtine and Bloch 2015).

More unexpectedly, all the participants regained voluntary control over the activity of previously paralyzed muscles without stimulation. This neurological recovery enabled the two less affected participants to walk overground with assistive devices in the absence of stimulation. One of the participants could even take a succession of independent steps between parallel bars. These results suggested that spatiotemporal neuromodulation protocols are not only important to promote a robust facilitation of locomotion, but may also play a critical role in steering activity-dependent plasticity in response to training. These protocols aim to increase the excitability of the motor neuron pools that are concomitantly modulated by task–specific sensory information and residual supraspinal command. This spatiotemporal convergence may trigger the reinforcement and growth of synaptic terminals from residual descending projections, as demonstrated in animal models (van den Brand et al. 2012; Asboth et al. 2018). This type of bidirectional spike–timing–dependent plasticity (Holtmaat and Svoboda 2009; Nishimura et al. 2013) has been observed consistently in humans with SCI (Perez et al. 2003; Urbin et al. 2017). Moreover, the repeated activation of proprioceptive afferents with EES may play an important role in promoting anatomical reorganization. Indeed, studies in mice demonstrated that proprioceptive afferents steer the reorganization of descending pathways that promotes a partial recovery of functions after SCI (Takeoka et al. 2014).

These combined studies have provided important proof of concept data on the ability of spinal cord neuromodulation therapies to raise the ceiling of recovery potential for patients with chronic SCI. However, this therapeutic strategy will likely be even more efficacious early after SCI, when the sudden damage has enhanced the potential for anatomical and functional reorganization and the neuromuscular system has not yet undergone the dramatic deterioration that follows chronic paralysis (Dietz 2010). Intervening in the early phase after SCI will require functional neurosurgeons to liaise effectively with neurologists, physical therapists, and engineers who can often operate in silos. It is important to point out that the recovery of supraspinal control over leg movements is directly correlated with the amount of spared tissues. More severe injuries would require the establishment of a digital bridge to control stimulation protocols, as summarized below.

### Brain-computer interface technologies

The original work from Evarts on the encoding of movement in the motor cortex (Evarts 1967) and from Fetz on the ability to train animals to control the activity of single neurons (Fetz 1969) paved the way towards brain computer interfaces (BCI). Accordingly, BCIs decode motor or cognitive intentions from neural recordings and translate these predictions into commands for computer programs or robotic arms (Gilja et al. 2015; Jarosiewicz et al. 2015). Implantable BCI technologies consist of intracortical microelectrode arrays (Utah arrays) that allow the recording of spiking activity, or electrode arrays positioned epidurally or subdurally over the cerebral cortex to monitor electrocorticogram signals (ECoG). Intracortical probes provide a high degree of spatial resolution (single neurons), but the signals tend to extinguish rapidly. Cortical grids allow more stable recordings but their spatial resolution may be insufficient for the most sophisticated prosthetic applications (Borton et al. 2013). The neural interfaces that have been used clinically are connected to a transdermal connector, which is not always well tolerated by patients and prone to infections. A survey of paralyzed patients demonstrated that they were twice as likely to adopt wireless technology compared to wired equivalents and that there were concerns around the aesthetic awkwardness of current BCI designs in addition to the chances of infection (Blabe et al. 2015). Various academic institutions and companies are developing wireless recording technologies that have already been validated in animal models (Yin et al. 2014; Mestais et al. 2015).

The most advanced BCI demonstrators have reached impressive levels of performance. Individuals with severe SCI have been able to operate biomimetic robotic arms (Hochberg et al. 2012; Collinger et al. 2013) to execute complex manual tasks using neural signals recorded from the primary motor cortex (M1). Moreover, encoding of touch pressure information into somatosensory cortex stimulation restored the ability to distinguish pressure-like sensations in each finger of the robotic hand (Flesher et al. 2016). Two individuals even learned to map M1 activity to neuromuscular stimulation programs in order to mobilize the upper limbs (Bouton et al. 2016; Ajiboye et al. 2017). The first patient used an array of 130 electrodes nested in a flexible sleeve wrapped around the arm. After 15 months of training, the patient was able to perform manual tasks requiring him to open his hand, perform a cylindrical palmar grasp and a precision pinch grasp (Bouton et al. 2016). In the second patient, 36 percutaneous electrodes were implanted into 18 muscles innervating the shoulder, elbow and hand. The patient was able to generate cortical commands to mobilize his arm in order to reach and drink from a mug and to feed himself (Ajiboye et al. 2017). Both studies provided important proof-of-concept data but also highlighted pragmatic issues that may preclude the rapid clinical dissemination of these BCIs. One of the key limitations was the difficulty to coordinate the direct recruitment of so many muscles in order to stabilize the posture of the arm and realize the tasks with fluidity.

BCI technologies have also been developed to restore leg movements (Fig. 1). Gait events such as the onset of the swing phase can be reliably decoded from M1 activity (Capogrosso et al. 2016; Bonizzato et al. 2018). These detections can trigger EES protocols that facilitate locomotor movements of the legs. Moreover, the cumulative firing of cortical ensemble populations can be linked to the intensity of the stimulation in order to determine the amplitude of leg movements. Rats with an SCI leading to leg paralysis were thus able to use this proportional brain-spine interface (BSI) to walk overground and accommodate leg movements to climb up a staircase (Bonizzato et al. 2018). This concept has successfully been translated into a BSI that restored locomotion in a non-human primate model of transient paralysis (Capogrosso et al. 2016). Intracortical microelectrode arrays were implanted in the leg area of M1. A wireless link mapped neural decoding of swing and stance events to EES protocols that promoted leg movements associated with these events. As early as 6 days post-lesion and without any prior training, this BSI restored weight-bearing locomotion of a paralyzed leg. In addition to the immediate recovery of leg movements, mounting evidence suggested that brain-actuated prostheses may augment training-mediated reorganization of nerve fibers (Bonizzato et al. 2018; Biasiucci et al. 2018; Donati et al. 2016). Rehabilitation programs closing the loop between circuits located above and below the injury may increase use–dependent neuroplasticity of residual connections through bidirectional spike–timing–dependent neuroplasticity (Ethier et al. 2015; Krucoff et al. 2016; McPherson et al. 2015). The neurological recovery observed in humans with SCI when rehabilitation is supported by spatiotemporal EES protocols may obey the same principles (see above) (Wagner et al. 2018). However, this interpretation remains speculative. More work is necessary to dissect the underlying mechanisms, and thus justify the surgical implantation of brain-spine interfaces in human patients. The computational complexity and skilled technological support may also need to be factored in prior to envisioning the clinical deployment of these neuroprostheses.

Electrode technologies are advancing rapidly, which may remedy some of the limitations of current probes. For example, the development of high-density silicone probes called Neuropixels has allowed the recording of approximately 100 neurons in freely moving mice (Stringer et al. 2019; Juavinett et al. 2018). The insertion of multiple Neuropixel probes enabled the simultaneous recording of thousands of neurons covering the visual and sensorimotor cortex, hippocampal formation, striatum, thalamus, and midbrain in mice (Stringer et al. 2019). This new technology has the potential to expand the number of brain regions that can be monitored in humans. Probe stiffness has been shown to damage to brain tissue and increase inflammation, which reduces signal stability and quality (Lacour et al. 2016). A new “sewing machine” system may remedy this issue: a single fine, stiff needle is used to insert many fine and flexible polymer electrodes into the brain (Hanson et al. 2019). This method ensures a maximal stiffness when penetrating brain tissue while maximizing flexibility and minimizing the size of the implant once inside the brain in order to reduce inflammation. Similarly, the soft implant termed electronic dura matter or e-dura can be inserted for extensive periods of time below the dura matter without causing significant inflammation (Minev et al. 2015). In rats, e-dura was surgically implanted over the motor cortex to monitor locomotor-related cortical activity, and over the spinal cord to deliver electrical and pharmacological stimulation that restored walking after paralysis. Improvements in electrode technologies are opening new avenues for improved recording and stimulation of the brain and spinal cord for patients with SCI.

#### Modulation of the spinal cord to regulate autonomic functions

EES has also demonstrated widespread benefits to autonomic systems including bowel and bladder function (Herrity et al. 2018; Walter et al. 2018) as well as the more extensively studied improvements in cardiovascular function (Aslan et al. 2018; Darrow et al. 2019; Harkema et al. 2018a; Harkema et al. 2018b; West et al. 2018) (Fig. 1). In the first case study, EES mediated immediate improvements in the blood pressure response to an orthostatic challenge and ameliorated the blood flow in the brain (West et al. 2018). The immediate ability of EES to stabilize blood pressure during an orthostatic challenge was then replicated (Darrow et al. 2019; Harkema et al. 2018a; Altaf et al. 2017). Moreover, the repeated application of EES protocols optimized for the modulation of blood pressure led to long-term improvements in cardiovascular regulation (Harkema et al. 2018b). These clinical observations are important, since improvements of cardiovascular functions are among the top health priorities for individuals with SCI (Anderson 2004) and a leading cause of death for this population (Garshick et al. 2005).

These results in patients with chronic SCI also raise the intriguing possibility to deliver EES during the sub-acute phase after injury. The maintenance of blood pressure during the first few days and weeks that follow an SCI is of particular clinical importance due to its significant volatility during this period. It is specifically this volatility that has spawned surgical teams to develop methods to optimize hemodynamic stabilization. The maintenance of spinal cord perfusion pressure contributes to predicting neurological recovery (Saadoun et al. n.d.; Squair et al. 2017). Currently, blood pressure is managed with noradrenergic and dopaminergic agonists, but these pharmacological agents are known to induce adverse events in the acute phase after injury (Altaf et al. 2017). Moreover, these slow-acting pharmacological agents cannot mitigate the bouts of severe hypo-perfusion that commonly occur in patients in the acute phase of SCI despite rigorous management of blood pressure (Kong et al. 2013). Bioelectronic implants may thus complement the arsenal of methods that are available to manage hemodynamics in the acute and sub-acute phase after an SCI and limit secondary complications such as autonomic dysreflexia.

The immediate increase in blood pressure in response to EES indicates that the activation of the sympathetic circuitry is driving the control of blood pressure. What remains unclear, however, is the mechanism by which EES delivered to the lumbar enlargement can modulate the sympathetic circuitry located within the thoracic spinal cord. It is therefore imperative to dissect the circuits through which EES modulate blood pressure. This knowledge is essential to operate a transition from empirical methods to evidence-based EES strategies that are optimized for blood pressure regulation. For example, the computational and physiological procedures that led to the development of spatiotemporal EES protocols (Wenger et al. 2016; Wenger et al. 2014; Formento et al. 2018) could be replicated to identify the optimal sites of stimulation and biologically-compliant EES protocols targeting the autonomic circuitry. The resulting conceptual and technological framework would not only lead to more effective treatments but would also guide neurosurgeons in the placement of the lead and configuration of stimulation protocols. Effectiveness and ease-of-use considerations are both pivotal for the widespread dissemination of bioelectronic treatments.

## Targeting circuits above the SCI: supralesional neuromodulation therapies

### Engaging hindbrain circuits involved in producing locomotion

Deep brain stimulation (DBS) of basal ganglia nuclei is a well-established treatment for movement disorders such as Parkinson’s disease, essential tremor, and inherited dystonias (Lozano and Lipsman 2013). DBS has also been used to modulate circuits above the SCI, but only in preclinical models. Studies conducted in rodent models have demonstrated that DBS delivered within the mesencephalic locomotor region (MLR) could improve locomotion SCI (Fig. 2). Historical studies conducted in Russia in the 1960s showed that electrical stimulation of this region engages reticulospinal neurons to trigger locomotion with a pace that is proportional to the stimulation amplitude (Ryczko and Dubuc 2013). Due to their distributed topology in the spinal cord, a fraction of reticulospinal fibers often survive the SCI, although they remain functionally silent when the lesion is severe (Asboth et al. 2018). The delivery of continuous electrical stimulation in the vicinity of the MLR immediately triggered walking in rats with such severe SCI (Bachmann et al. 2013). Increasing the intensity of stimulation resulted in greater walking speed and high step frequency. The stimulation also increased the range of leg motion and reduced the amount of paw dragging. The MLR is functionally equivalent to the pedunculopontine nucleus (PPN) region in humans. DBS delivered in the PPN in humans with Parkinson’s disease has reduced freezing of gait and falls, albeit results have been variable (Stefani et al. 2007; Tsang et al. 2010). A phase one clinical trial has been approved in the Spinal Cord Injury Center Balgrist to test this approach in five patients with partial SCI (https://clinicaltrials.gov/ct2/show/NCT03053791).

The nucleus raphe magnus (NRM) has also been targeted with electrical stimulation in rodent models of SCI in order to augment the release of serotonin. Indeed, this region is the main source of serotonin to the spinal cord (Jordan et al. 2008). In one study, 5 min of 8 Hz stimulation alternated with 5 min of rest for 12 h during the day with 12 h of nocturnal rest was applied chronically after a mid-thoracic contusion SCI (Hentall and Burns 2009). NRM stimulation was found to reverse forepaw allodynia at 6 weeks after injury. However, there was no difference between the stimulated and non-stimulated groups in terms of lesion cavity size, volume of contusion, and on neuronal preservation although there was reduced astroglial scar formation (Hentall and Burns 2009).

Compared to the extensive literature on the impact of SCI on spinal circuits and descending projections within the spinal cord, there is a paucity of studies that investigated SCI-related changes in brain circuit dynamics, and how specific circuits contribute to steering recovery after SCI. However, there is an increasing understanding that the brain is critically needed to cure SCI (Sawada et al. 2015; Isa 2017). As researchers continue dissecting circuit properties following spinal cord damage, novel targets might be discovered to improve functional recovery with neuromodulation therapies delivered within supraspinal structures.

### Augmenting circuit reorganization with vagal nerve stimulation

Another area of neuromodulation that has received attention is vagal nerve stimulation (VNS) (Fig. 2). Previous research has demonstrated that the precise temporal pairing of vagal nerve stimulation with movement execution can improve motor recovery in rodent models of stroke (Hulsey et al. 2016; Khodaparast et al. 2014; Khodaparast et al. 2016). VNS is thought to lead to the release of monoamines within the cerebral cortex, which may promote plasticity of neural circuits and enhance motor learning (Hulsey et al. 2016). Based on these encouraging results, this strategy was tested in rodent models of unilateral cervical contusion (C6) (Ganzer et al. 2018). Rats were trained to retrieve food reward with their forepaw. Each successful grasp was followed by an electrical burst delivered to the VNS via a bipolar cuff electrode implanted around the left cervical vagus nerve. VNS resulted in significantly improved reaching force compared to rehabilitation alone. The temporal contingence between VNS and the executed movement was critical to promote the recovery. Anatomical and electrophysiological experiments showed that this rehabilitation paradigm enhanced the reorganization of cortical circuits and promoted the growth of new corticospinal tract projections within the cervical spinal cord (Ganzer et al. 2018). Due to its broad functional connectome, the vagal nerve augments the activity of various sensorimotor and autonomic systems. Therefore, VNS likely increases the level of activity within the circuits that are also contributing to movement execution—thus engaging activity-dependent plasticity rules (Edgerton and Gad 2018).

### Augmenting circuit reorganization with cortical surface stimulation

Activity leads to the functional and anatomical reinforcement of the repeatedly activated neural connections (Edgerton et al. 2004; Raineteau and Schwab 2001; Cote et al. 2017; Torres-Espin et al. 2018). These well-known physiological principles fostered the development of stimulation paradigms that aim to enhance the activity of neurons with residual neural projections in the spinal cord after SCI. The goal was to promote the growth of new connections in order to improve functional recovery. For instance, electrical motor cortex stimulation has been shown to mediate robust sprouting of spared corticospinal tract fibers. This anatomical reorganization has been associated with improvement of skilled locomotion in rodent models of SCI (Carmel and Martin 2014; Zareen et al. 2017). In this scenario, the stimulation was applied continuously for many hours per day. However, previous studies using spinal cord or vagal nerve stimulation showed that pairing the stimulation with movement execution during rehabilitation may further augment the impact of this treatment (Ganzer et al. 2018).

Similar principles have been applied in humans with SCI using noninvasive technologies. For example, transcranial magnetic stimulation (TMS) applied over the human motor cortex augmented the transmission along descending neural pathways. This increase in conductivity improved motor functions and reduced spasticity (Tazoe and Perez 2015; Long et al. 2017). Along the same vein, paired associative stimulation of the motor cortex and reflex circuits located below the SCI durably augmented the efficacy of the recruited circuits (Mishra et al. 2017; Dixon et al. 2016). These approaches may increase recovery after partial SCI that spare corticospinal tract projections. Neurotechnologies for chronic electrical motor cortex stimulation are available for clinical use in humans. We thus anticipate that clinical trials may test the efficacy of these bioelectronic treatment paradigms to augment functional recovery in humans with SCI.

## New role for functional neurosurgery in SCI medicine

The role of the functional neurosurgeon in SCI medicine is currently restricted to the occasional treatment of spasticity or chronic pain in the chronic stage of SCI. Acute treatments are usually performed by spine surgeons. The advent of bioelectronic technologies will transform the role of functional neurosurgeons in spinal pathology (Borton et al. 2013). The flurry of advances in SCI-related bioelectronic medicine is opening unprecedented opportunities to impact the neurological recovery and quality of life of patients with SCI. Obviously, functional neurosurgeons will be in charge of the precise implantation of stimulating and recording neural interfaces over the spinal cord or within the brain; together with active electronics. The pre-operative identification of the optimal implant location and intraoperative guidance for inserting and securing implants will require interactions with neural engineers and healthcare professionals who will also follow the patients post-operatively. Indeed, critical to SCI-related bioelectronic treatments is the need for extensive tuning of the therapies post-operatively during long-lasting and highly personalized rehabilitation programs. This specificity may require more sustained involvement by functional neurosurgeons in the deployment of the treatments. They will have to maintain constant interactions with interventional neurologists who will intervene in the neurological recovery of their patients. These interventional neurologists will dialogue with the functional neurosurgeons and rehabilitation teams to identify the optimal treatment options based on the current neurological status, functional needs, and recovery potential of each patient at each relevant time-point, asking questions such as: Is hemodynamic stabilization critical at this time point? Is there potential for increasing neurological recovery with neurotechnologies that enable active motor rehabilitation? Can we anticipate increased anatomical reorganization of neuronal connections with chronic modulation of the brain regions containing neurons with spared projections in the spinal cord? Is there a potential benefit to provide a BCI treatment to enable the control of computers or robotic arms with brain signals, and thus to improve interactions with the environment? Many questions and opportunities will thus open a new dialogue in neurorestorative interventional medicine and neuroprosthetics. Finally, we anticipate that this bioelectronic medicine revolution will not be limited to SCI, but will quickly expand to other fields such as traumatic brain injury, stroke, and neurodegenerative disorders.

## Conclusions

SCI remains a challenging disease to treat. Despite having significant impacts on lives of patients across the world, years of research into improving neurologic outcomes after injury have yet to find a cure. Relatively recently, there has been a surge in bioelectronic technological developments including spatiotemporal epidural spinal stimulators, brain-spine interfaces, and deep brain stimulation paradigms for various locomotor diseases including SCI. With these developments, there have been clinical improvements in human SCI patients never seen before. The potential promise of these new technologies for SCI has significant implications for clinicians treating SCI patients, especially neurosurgeons. Traditionally, spinal surgeons have been at the forefront of generating guidelines for spinal trauma. However, the increasing involvement of functional neurosurgery in treating SCI will likely parallel the development of new technologies for improving function after SCI. As bioelectronic technologies continue to advance, close collaboration and dialogue between multiple professions including surgeons, neurologists, and engineers will be a necessity more than ever before.

## Data Availability

Not applicable.

## Abbreviations

BCI: Brain-computer interface
BSI: Brain-spine interface
CSF: Cerebrospinal fluid
DBS: Deep brain stimulation
ECoG: Electrocorticogram
EES: Epidural electrical stimulation
FEM: Finite element modelling
IMU: Inertial measurement units
M1: Primary motor cortex
MLR: Mesencephalic locomotor region
MRI: Magnetic resonance imaging
NRM: Nucleus raphe magnus
PPN: Pedunculopontine nucleus
SCI: Spinal cord injury
TMS: Transcranial magnetic stimulation
VNS: Vagal nerve stimulation
